# Smoking on the risk of acute respiratory distress syndrome: a systematic review and meta-analysis

**DOI:** 10.1186/s13054-024-04902-6

**Published:** 2024-04-14

**Authors:** Lujia Zhang, Jiahuan Xu, Yue Li, Fanqi Meng, Wei Wang

**Affiliations:** 1 Institute of Respiratory and Critical Care Medicine, The First Hospital of China Medical University, No. 155 Nanjing North Street, Heping District, Shenyang, 110001 Liaoning China; 2 Institute of Respiratory and Critical Care Medicine, First Affiliated Hospital of Dalian Medical University, Dalian, Liaoning China

**Keywords:** Acute respiratory distress syndrome, Smoking, Meta-analysis, Systematic review

## Abstract

**Background:**

The relationship between smoking and the risk of acute respiratory distress syndrome (ARDS) has been recognized, but the conclusions have been inconsistent. This systematic review and meta-analysis investigated the association between smoking and ARDS risk in adults.

**Methods:**

The PubMed, EMBASE, Cochrane Library, and Web of Science databases were searched for eligible studies published from January 1, 2000, to December 31, 2023. We enrolled adult patients exhibiting clinical risk factors for ARDS and smoking condition. Outcomes were quantified using odds ratios (ORs) for binary variables and mean differences (MDs) for continuous variables, with a standard 95% confidence interval (CI).

**Results:**

A total of 26 observational studies involving 36,995 patients were included. The meta-analysis revealed a significant association between smoking and an increased risk of ARDS (OR 1.67; 95% CI 1.33–2.08; *P* < 0.001). Further analysis revealed that the associations between patient-reported smoking history and ARDS occurrence were generally similar to the results of all the studies (OR 1.78; 95% CI 1.38–2.28; *P* < 0.001). In contrast, patients identified through the detection of tobacco metabolites (cotinine, a metabolite of nicotine, and 4-(methylnitrosamino)-1-(3-pyridyl)-1-butanol (NNAL), a metabolite of tobacco products) showed no significant difference in ARDS risk (OR 1.19; 95% CI 0.69–2.05; *P* = 0.53). The smoking group was younger than the control group (MD − 7.15; 95% CI − 11.58 to − 2.72; *P* = 0.002). Subgroup analysis revealed that smoking notably elevated the incidence of ARDS with extrapulmonary etiologies (OR 1.85; 95% CI 1.43–2.38; *P* < 0.001). Publication bias did not affect the integrity of our conclusions. Sensitivity analysis further reinforced the reliability of our aggregated outcomes.

**Conclusions:**

There is a strong association between smoking and elevated ARDS risk. This emphasizes the need for thorough assessment of patients' smoking status, urging healthcare providers to vigilantly monitor individuals with a history of smoking, especially those with additional extrapulmonary risk factors for ARDS.

**Supplementary Information:**

The online version contains supplementary material available at 10.1186/s13054-024-04902-6.

## Introduction

ARDS is a prevalent clinical syndrome, characterized by acute respiratory failure resulting from diffuse pulmonary inflammation and edema. Characterized by hypoxemia and evident changes in lung imaging via chest X-rays or CT scans, ARDS is associated with a high incidence and mortality [1]. ARDS constitutes a significant health burden globally, accounting for 10.4% of ICU admissions, and approximately 23% of these patients necessitate mechanical ventilation [2]. Despite numerous advancements in treatment, the mortality rate of ARDS remains around 30% as of 2021 [3]. Consequently, emphasis has shifted toward ARDS prevention, highlighting the need to identify and manage modifiable risk factors to decrease its incidence.

The clinical precursors to ARDS encompass a range of clinical conditions including sepsis, pneumonia, aspiration, trauma, surgical procedures, pancreatitis, transfusions, and inhalation injuries from smoke or toxic gases [4]. Despite this understanding, there is still a limited grasp of the environmental factors contributing to ARDS, highlighting the need for additional research in this area [5]. A pivotal 1996 cohort study initially linked smoking with an elevated risk of ARDS [6]. Subsequent research on various patient demographics has underscored this connection, though with inconsistent findings. A 2014 narrative review identified smoking as a key environmental risk factor for ARDS after analyzing three studies [6–9]. However, robust epidemiological evidence linking smoking to ARDS risk remains sparse [10]. Our systematic review and meta-analysis of observational studies aimed to consolidate recent findings, elucidating the relationship between smoking and ARDS risk.

## Methods

### Protocol and registration

This systematic review and meta-analysis adhered to the PRISMA guidelines (http://www.prisma-statement.org/) and was registered on PROSPERO (CRD42023483876). (Additional file 1: Table S1).

### Search strategy

We conducted a comprehensive search of PubMed, EMBASE, the Cochrane Library, and Web of Science for studies published between 2000 and 2023 without language restrictions. The search terms used included all possible combinations of ARDS and smoking. A copy of our search strategy could be found in our online Supplementary Materials (Additional file 1: Table S2). We screened the reference lists of relevant systematic reviews and meta-analyses to ensure that we did not miss any additional articles.

### Study selection criteria

#### Participants

Patients aged 18 or older with clinical risk factors for ARDS were included.

#### Exposure

We included studies about assessing smoking via clinical records or laboratory tests. The smoking history (including smokers, nonsmokers, current smokers, former smokers, and never smokers) was extracted from clinical records. The laboratory tests included the detection of tobacco product metabolites, such as serum cotinine (nonsmokers < 0.02 ng/ml, passive smokers 0.02–3.08 ng/ml, active smokers > 3.08 ng/ml) and urine NNAL (nonsmokers < 0.25 pg/mg, passive smokers 0.25–47 pg/mg, active smokers > 47 pg/mg) [11].

#### Outcomes

Studies with outcomes for ARDS were included. Studies limited to other diagnoses were excluded.

#### Study design

We included observational cohort and case‒control studies; excluded reviews, animal and cell studies, abstracts, comments, case reports, and cross-sectional studies. In case of duplicated data, the study with more complete and detailed data was chosen. We also excluded studies in which a 2 × 2 table between exposure to smoking and the outcome of ARDS could not be constructed. Two independent reviewers (LJZ and JHX) screened the titles and abstracts, and the full texts of the studies that met the inclusion criteria were obtained. Any disagreements encountered during the screening process were resolved through discussion and, if necessary, with the assistance of a third reviewer (YL).

### Data extraction

The data were independently extracted by two reviewers (LJZ and JHX) with electronic spreadsheets. The extracted information included the first author's name, publication year, patient data source, ARDS diagnostic criteria, sample size, clinical risk factors for ARDS, methods of obtaining smoking status, adjustments for confounding factors, smoking status, incidence rates of ARDS, age, injury severity scores (ISSs), and mortality rates. Smoking status was categorized into two groups: smokers (experimental group) and nonsmokers (control group). In the analysis, current smokers, former smokers, active smokers, and passive smokers were all categorized into the smoking group. The method for unknown non-normal distributions was applied to transform the median and quartile into the mean and standard deviation (SD), respectively. Graphical data from the original studies were extracted by using WebPlotDigitizer, a semiautomated tool that extracts underlying digital data by reverse engineering a visual image of the data. The extraction forms were compared, and disagreements were resolved firstly by discussion or with a third reviewer (YL) if a consensus could not be reached.

### Quality assessment

The quality of the studies was assessed by the Newcastle–Ottawa Scale. High quality was defined as a grade > 6. Both cohort and case‒control studies had a maximum score of 9. Two independent reviewers assessed the quality of the included studies, and any disagreements were resolved through discussion.

### Statistical analysis

Data synthesis was conducted with Review Manager software version 5.4 (Cochrane Collaboration). We reported ORs for dichotomous outcomes and MDs for continuous outcomes, with a standard 95% CI. To assess heterogeneity among the studies, we employed the Cochrane Q test and used the *I*^2^ statistic to measure the degree of heterogeneity. High heterogeneity was inferred at *I*^2^ > 50%, prompting the use of a random-effects model; otherwise, a fixed-effects model was applied. Subgroup analysis focused on clinical risk factors, assessment methods of smoking status, and diagnostic criteria of ARDS. Publication bias was evaluated by using Egger's test, and complemented by visual inspection through funnel plots. We conducted sensitivity analyses using two approaches. Firstly, we excluded individual studies sequentially to assess their individual impact on the overall results. Secondly, in a similar manner, we excluded different subgroups of clinical risk factors to gauge their specific effects on the stability of our findings. Publication bias and sensitivity analyses were conducted with Stata/MP 17.0 (StataCorp LLC, Texas, US). A *P* value < 0.05 indicated statistical significance.

## Results

### Literature search process

**Fig. 1:**
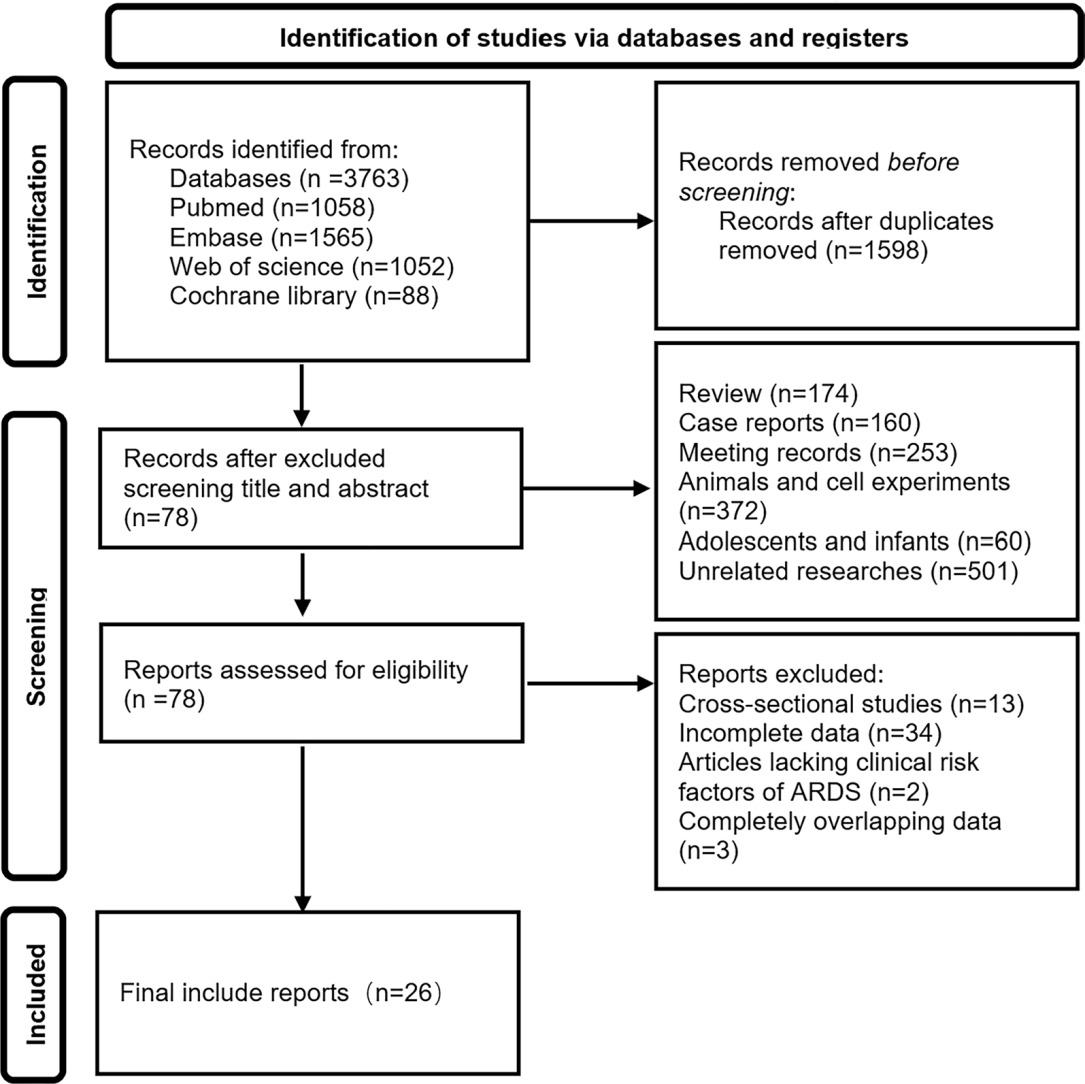
Preferred reporting items for systematic reviews and meta-analyses (PRISMA) chart. The process of literature selection

Database searches and reference lists yielded a total of 3763 articles. After the removal of duplicates, we identified 1598 articles for title and abstract screening, from which we identified 78 articles for full text review. Ultimately, 26 of these articles met the inclusion and exclusion criteria. The process of literature selection is detailed in Fig. 1.

### Study characteristics

This review included 26 studies involving a total of 36,995 participants; the results are detailed in Table 1. The majority (25 studies) [10, 12–30, 32–36] were cohort studies, and one was a case‒control study [31]. All the studies were conducted under hospital circumstances. Quality assessment using the Newcastle‒Ottawa Scale revealed that all 26 studies were of medium to high quality. Information on smoking history was extracted from 22 studies to assess the relationship between smoking and ARDS, while four studies assessed patients' exposure to smoking through the detection of tobacco product metabolites. Various ARDS diagnostic criteria were used, including the Berlin definition, the American-European Consensus Conference definition (AECC), the International Classification of Disease-10 standards (ICD-10), the fifth edition of the Indonesian COVID-19 guidelines, and criteria set by the Chinese Medical Association's Critical Care Medicine Branch in 2006.

**Table 1:** The characteristics of the included studies

Study	Year	Main predisposing condition	Definition used to ascertain ARDS	No. of people include	Clinical risk factor	Assessment of smoking	Adjustment	Quality
AlleaBelle Gongola	2021	Trauma center	Unnoticed	1880	After trauma	Smoke history	Age, sex, race, and alcohol use disorder	7
Ariane R. Panzer	2018	ICU	The Berlin Criteria	74	After trauma	Plasma cotinine	gender	6
B. Dai	2010	Hospitalized patients	Criteria set by the Chinese Medical Association's Critical Care Medicine Branch in 2006	92	Virus pneumonia	Smoke history	No adjustment	5
Benjamin M. Aakre	2014	Hospitalized patients	the Berlin Criteria	316	After surgery	Smoke history	Age	6
Carolyn S. Calfee	2015	ICU	AECC	426	Sepsis, pneumonia, aspiration, pancreatitis, near drowning, drug overdose, or other	Urine NNAL, smoke history	Age, gender, alcohol abuse	7
Emma Rachmawati	2021	Hospitalized patients	The fifth edition of the Indonesian COVID-19 guidelines	490	Covid-19	Smoke history	No adjustment	4
Farzad Moazed	2020	ICU	The Berlin Criteria	635	After trauma	Plasma cotinine	BMI, ISS	7
Farzad Moazed	2022	ICU	Both AECC and the Berlin Criteria	592	Sepsis	Plasma cotinine, Urine NNAL	APACHE II, Vasopressor use	7
H. Lei	2013	Hospitalized patients	AECC	184	Severe acute pancreatitis	Smoke history	No adjustment	4
Hiroki Iriyama	2020	ICU	The Berlin Criteria	549	Non-pulmonary sepsis	Smoke history	Age, gender	6
J. Elmer	2013	Hospital database	AECC	697	After spontaneous intracerebral hemorrhage	Smoke history	No adjustment	5
Jun Ying	2019	RICU	The Berlin Criteria	145	Severe Pneumonia	Smoke history	No adjustment	5
Majid Afshar MD MSc	2017	Burn center	The Berlin Criteria	2485	After burn-injured	Smoke history	Age, sex, mechanism of burn, total body surface area injured, and inhalation injury	7
Mats Christian Højbjerg Lassen	2021	Hospitalized patients	The Berlin Criteria	168	Covid-19	Smoke history	Age, BMI, gender	6
Ognjen Gajic	2011	Emergency department	AECC	5204	High-risk trauma, high-risk surgery, aspiration, sepsis, shock, pneumonia, and pancreatitis	Smoke history	Age	6
P. Wacharasint	2016	SICU	AECC	4652	After surgery	Smoke history	Age, gender, operated or non-operated, APACHE II score, and patient’s pre-existing diseases	8
Paul Balfanz	2021	ICU	The Berlin Criteria	125	Covid-19	Smoking history	No adjustment	5
Phillip A Howells	2017	Hospitalized patients	The Berlin Criteria	129	After surgery	Smoke history	Age	5
S. Sang	2021	Hospitalized patients	ICD-10	16,509	Pneumonia and/or influenza	Smoke history	No adjustment	5
Shilei Li	2020	Hospitalized patients	The Berlin Criteria	150	Sepsis	Smoke history	No adjustment	5
T. N. Ferro	2010	Trauma center	AECC	327	After trauma	Smoke history	No adjustment	5
W. Chen	2020	Hospitalized patients	The Berlin Criteria	104	Sepsis	Smoke history	No adjustment	4
X. Huang	2019	ICU	The Berlin Criteria	152	Sepsis	Smoke history	No adjustment	6
Y. Matusov	2020	Trauma center	The Berlin Criteria	272	Sepsis	Smoke history	No adjustment	5
Y. Wang	2019	ICU	The Berlin Criteria	109	Sepsis	Smoke history	No adjustment	6
Yuequan Shi	2022	ICU	The Berlin Criteria	529	Sepsis and pneumonia	Smoke history	age, gender	6

ICU, intensive care unit; AECC, American-European Consensus Conference definition; BMI, body mass index; APACHE II, Acute physiology and chronic health evaluation II; RICU, respiratory intensive care unit; SICU, surgical intensive care unit; ICD-10, the international classification of disease-10

### Meta-analysis

#### Smoking and ARDS risk

**Fig. 2:**
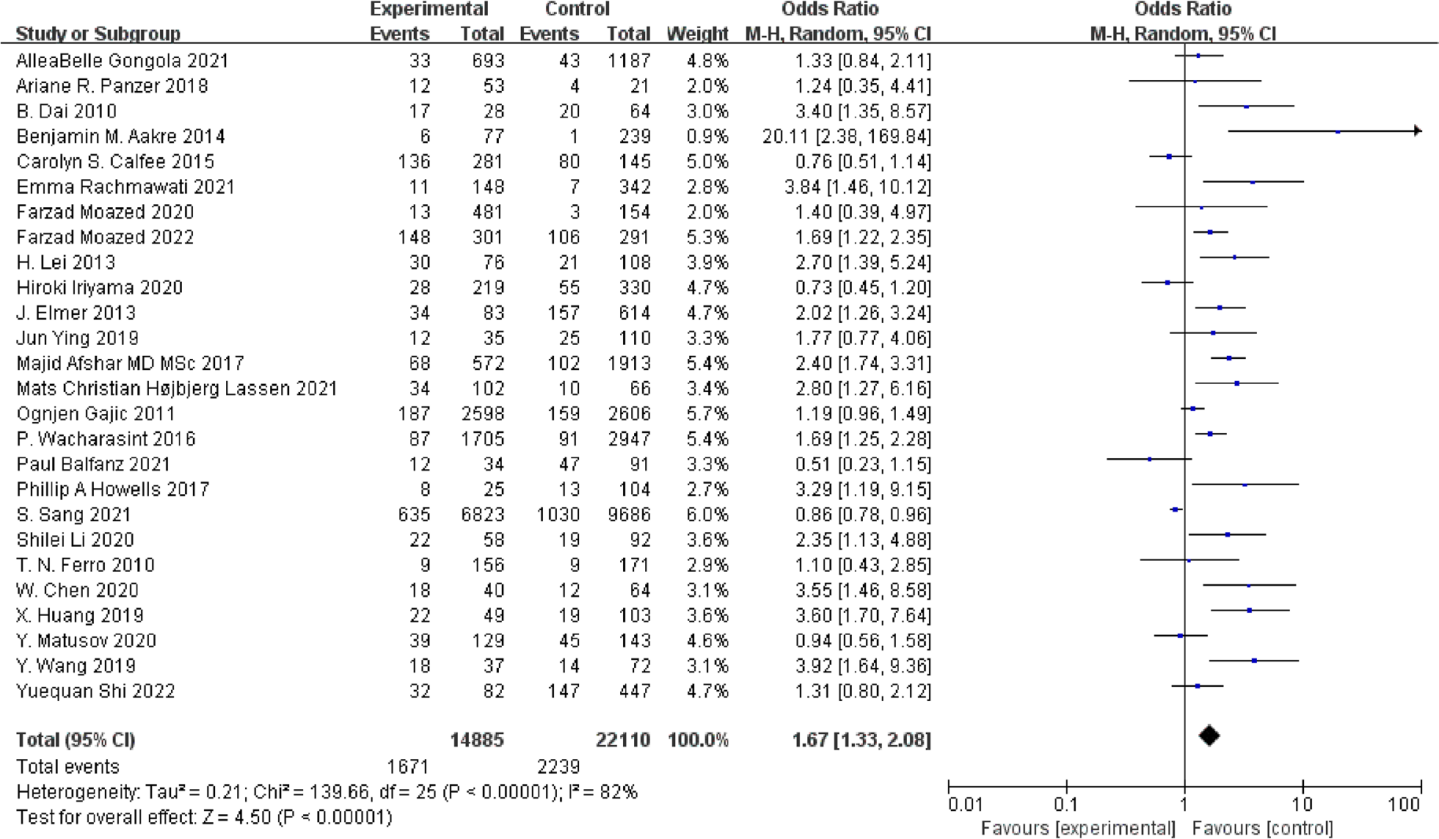
ORs of ARDS between smokers and nonsmokers

Analysis of 26 studies revealed a significant association between smoking and increased ARDS risk (OR 1.67; 95% CI 1.33–2.08; *P* < 0.001) (Fig. 2).

#### Smoking status assessment methods and ARDS risk

**Fig. 3:**
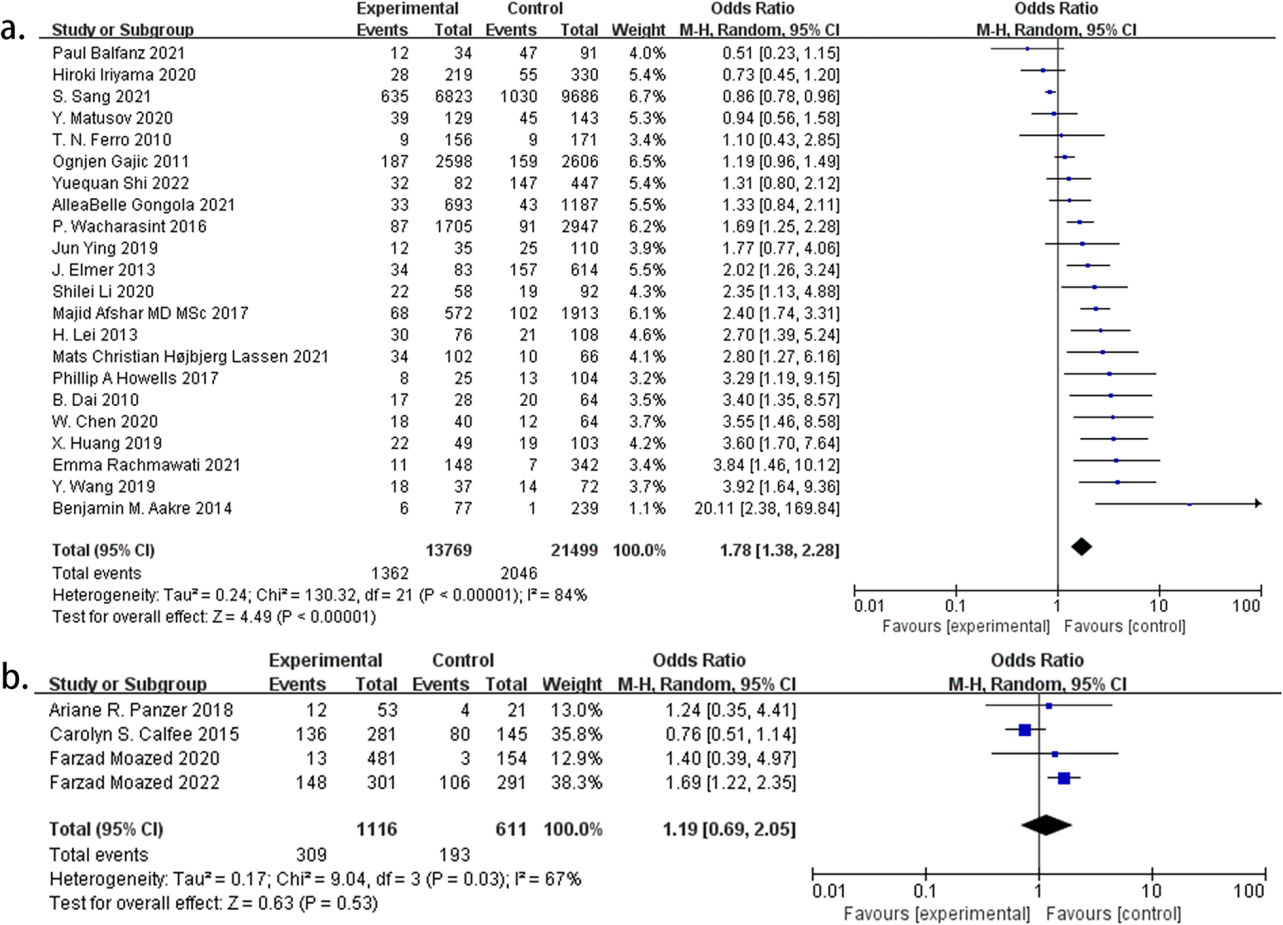
**a** ORs of ARDS between smokers and nonsmokers assessed by smoking history. **b** ORs of ARDS between smokers and nonsmokers assessed by tobacco metabolites

In 22 studies, ARDS risk was evaluated based on smoking history, and the results showed a substantial increase in risk generally similar to that reported in all the studies (OR 1.78; 95% CI 1.38–2.28; *P* < 0.001). Conversely, four studies utilizing tobacco metabolite detection did not show a notable increase in risk (OR 1.19; 95% CI 0.69–2.05; *P* = 0.53) (Fig. 3).

#### Smoking cessation and ARDS risk

**Fig. 4:**
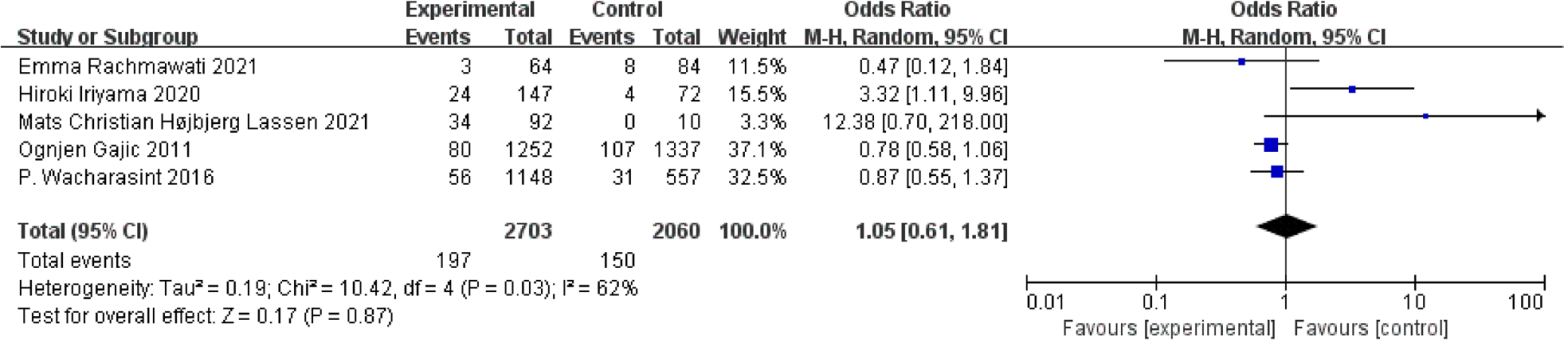
ORs of ARDS between current and former smokers

Among the five studies differentiating current smokers from former smokers, smoking cessation did not significantly reduce ARDS risk (OR 1.05; 95% CI 0.61–1.81; *P* = 0.87) (Fig. 4).

#### Characteristics of the smoking population in ARDS risk studies

**Fig. 5:**
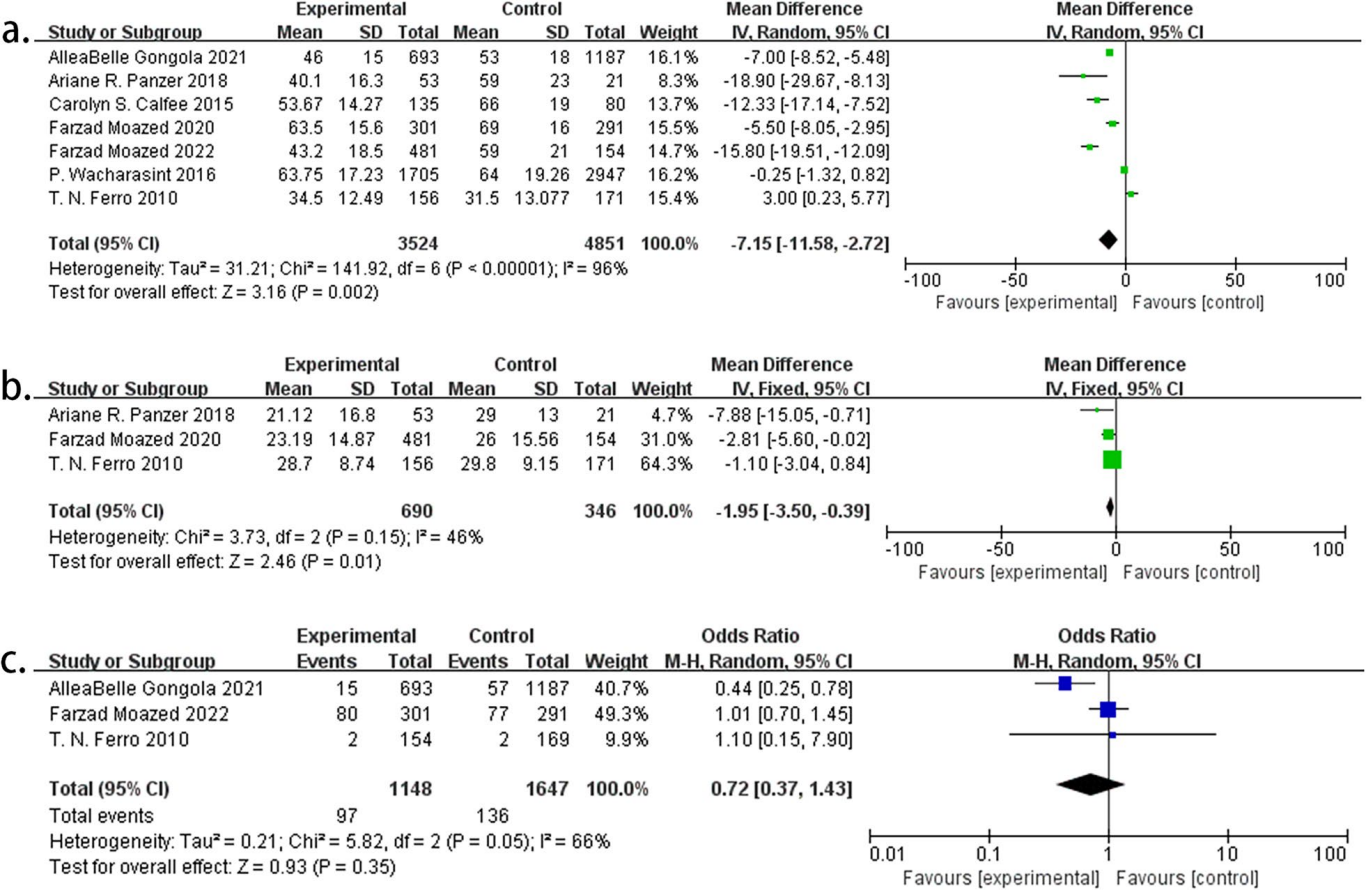
**a** MDs of age between smokers and nonsmokers. **b** MDs of the ISSs between smokers and nonsmokers in trauma cohorts. **c** MDs of mortality between smokers and nonsmokers

Seven studies presented age data, highlighting that smokers in populations with clinical risk factors for ARDS were younger than nonsmokers (MD − 7.15; 95% CI − 11.58 to − 2.72; *P* = 0.002). Among the trauma cohort studies, three provided ISS data for smoking and nonsmoking populations, indicating that smokers had lower ISSs (MD − 1.95; 95% CI − 3.50 to − 0.39; *P* = 0.01). Additionally, three studies compared mortality rates between smoking and nonsmoking populations within the same group and revealed no significant difference (MD 0.72; 95% CI 0.37–1.43; *P* = 0.35) (Fig. 5).

#### Subgroup analysis

**Fig. 6:**
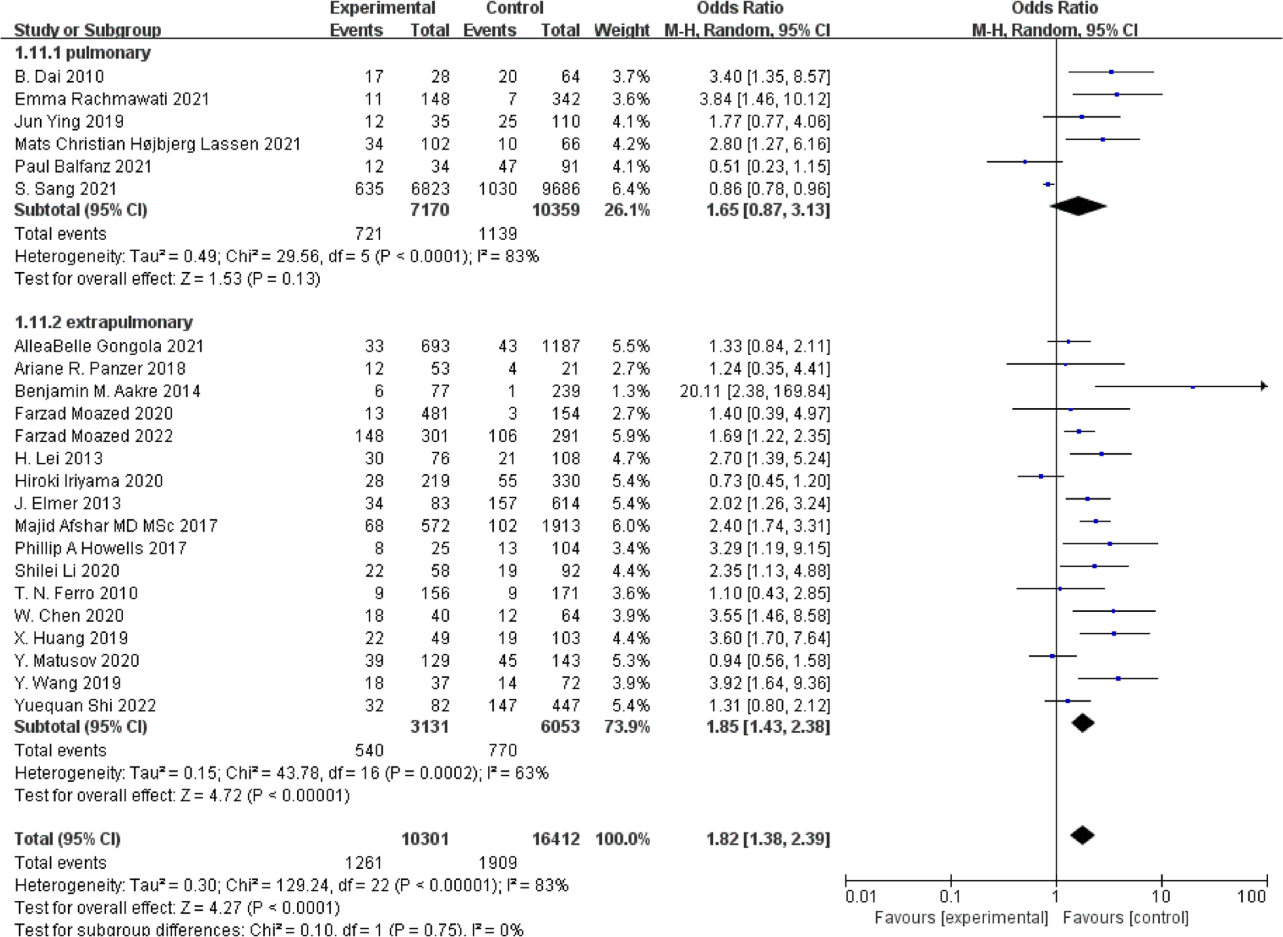
Subgroup analysis, comparing pulmonary and extrapulmonary risk factors

By analyzing subgroups based on clinical risk factors, the study revealed a notably greater risk of ARDS in patients with sepsis, trauma or burns, and surgery (OR 1.75; 95% CI 1.17–2.62; *P* = 0.006 vs. OR 1.66; 95% CI 1.15–2.40; *P* = 0.007 vs. OR 6.37; 95% CI 1.12–36.13; *P* = 0.04) (Additional file 1: Fig. S1). When the pathogenic factors were divided into pulmonary (COVID-19, other pneumonias) and extrapulmonary (sepsis, trauma burns, surgery, acute pancreatitis, postcerebral hemorrhage) categories, smoking was significantly linked to an increased risk of ARDS from extrapulmonary causes (pulmonary factors OR 1.65; 95% CI 0.87–3.13; *P* = 0.13 vs. extrapulmonary factors OR 1.85; 95% CI 1.43–2.38; *P* < 0.001) (Fig. 6).

Subgroup analyses based on ARDS diagnostic criteria and assessment methods of smoking status showed no significant difference between subgroups with low heterogeneity (Additional file 1: Figs. S4, S5).

### Publication bias

Asymmetry in the funnel plot indicated potential publication bias. However, a trim and fill analysis of studies located on the left side of the funnel plot resulted in an OR of 1.442 (95% CI 1.167–1.782), suggesting that this bias did not substantially impact the overall findings' robustness (Additional file 1: Fig. S2).

### Sensitivity analysis

The sensitivity analysis, conducted due to substantial heterogeneity (*I*^2^ = 82% > 50%), validated the stability of the meta-analysis results. The analysis showed that removing individual studies and each subgroup of clinical risk factors, independently, did not markedly alter the overall conclusions, thereby ensured the reliability of the combined data (Additional file 1: Fig. S3, Table S3).

## Discussion

Adding value to this study, this is the first and most comprehensive meta-analysis focusing on the association between smoking and the risk of ARDS among observational studies. This study critically evaluated various research evidence and examined whether this risk could change with different ARDS pathogenesis factors. This highlighted how smoking significantly elevated the risk of ARDS, categorizing it as a notable environmental risk factor. The analysis particularly underscored the heightened risk in patients involving extrapulmonary pathogenic factors such as sepsis and trauma. Intriguingly, smoking increased ARDS risk even in younger patients with less severe pathogenic injuries. Furthermore, smoking cessation could not substantially alter the elevated risk, suggesting that the adverse impact of smoking on lung health was deeper and more lasting than previously understood.

Cigarette smoke, a complex mixture of gases and particles, exhibits oxidative, proinflammatory, and carcinogenic properties [37]. This composition adversely affects lung function through multiple mechanisms, such as elevating alveolar epithelial permeability, disrupting normal immune responses, and causing vascular endothelial damage, all of which are critical pathological and physiological mechanisms of ARDS [7, 38]. Research indicated that smoking-induced pathological alterations in the alveolar epithelium mirrored those observed in ARDS. Studies in cells and animals revealed that oxidative stress caused by cigarette smoke could negatively affect the integrity of alveolar epithelial cell connections and suppress the expression of alveolar ion channels [39, 40]. This damage could increase the likelihood of severe edema in the lungs when the patient was exposed to ARDS clinical risk factors. Additionally, smoking directly impacted both innate and adaptive immunity, potentially leading to a heightened risk of infections [41]. These effects included impaired mucociliary function, reduced clearance of bacteria by alveolar macrophages, decreased surfactant production, altered T-cell responses, NK cell function suppression, and decreased immunoglobulin levels [38, 42]. Smoking triggered the influx of neutrophils into the tissues surrounding blood vessels. These neutrophils, along with activated macrophages, released inflammatory cytokines and proteolytic enzymes, contributing to the inflammatory response [43]. Additionally, smoking facilitated biofilm formation, enhancing pathogen virulence, which played a critical role in escalating the risk of respiratory infections [44]. These impaired immune defenses and increased infection susceptibility constituted another potential mechanism through which smoking increased the risk of ARDS. Smoking was also known to cause endothelial injury and alter endothelial functions [45]. Oxidative stress caused by cigarette smoke could change the cytoskeletal structure of endothelial cells, leading to dysfunction of the endothelial barrier. In vitro studies showed that cigarette smoke extract exacerbated the endothelial dysfunction caused by lipopolysaccharides [46]. Moreover, smoking could enhance platelet activation, thereby exacerbating endothelial cell damage and triggering thrombosis [47]. The resulting pulmonary artery thrombosis contributed to microvascular filling defects, a key factor in abnormal gas exchange in ARDS patients. The intricate connection between smoking and ARDS still was an area of ongoing investigation. This uncertainty presented challenges in the development of effective treatment targets, highlighting the need for additional foundational research in this area.

As a clinical syndrome, ARDS arises from various causes, leading to a common pathological result: protein-rich pulmonary edema [3]. However, ample evidence suggested that ARDS caused by different etiologies exhibited heterogeneity in its pathological process. Both pulmonary and extrapulmonary risk factors were able to trigger ARDS, with each category influencing the disease's development and characteristics, respectively. Direct pulmonary injury-induced ARDS typically led to extensive damage and inflammation in the alveolar epithelium [48]. In patients with ARDS triggered by extrapulmonary risk factors, the damage extended beyond the lungs, often leading to severe endothelial injury and systemic inflammation. This form of ARDS was closely linked to endothelial dysfunction, where a compromised endothelial barrier allowed systemic inflammation to spread into the lungs. Consequently, this led to heightened alveolar epithelial inflammation and an exaggerated macrophage inflammatory response, playing a pivotal role in the manifestation of ARDS [1]. Taken together, our findings highlighted that smoking predominantly escalated the risk of ARDS stemming from extrapulmonary risk factors. These findings suggested that the impairment of endothelial barrier function, a consequence of smoking, could be a key factor in elevating ARDS risk. Emphasizing the reinforcement of endothelial barrier functionality was pivotal for the prevention of ARDS in patients with extrapulmonary causes, particularly in individuals who smoked. Such a focus could play a crucial role in diminishing the occurrence of ARDS in these susceptible populations.

Aging serves as both a risk factor for ARDS and a determinant of the severity of lung injury. However, our study revealed that within the same high-risk group for ARDS, the smoking population could tend to be younger. A multicenter study of ARDS patients exposed to cigarette smoke showed that younger smokers, despite having fewer instances of septic shock and better overall health, had similar levels of lung injury as older nonsmokers did [18, 49]. These findings suggested that younger, otherwise healthy individuals who smoke, were more susceptible to ARDS. The elevated risk of ARDS associated with aging could be attributed to a decrease in the body's defense mechanisms; a greater incidence of diseases such as pneumonia and overactivation of inflammatory pathways such as NF-κB [50]. Research findings indicated that neutrophils from elderly individuals exhibited nontargeted tissue migration, increasing release of primary granules, elevating neutrophil elastase activity, and led to heightened tissue inflammation [51]. These cellular changes contributed to a more inflammatory internal state in older adults, escalating their vulnerability to intense inflammatory reactions in response to lung injuries. A systematic review of animal studies also revealed a correlation between increased age and more severe lung injuries [52]. Basic research demonstrated that cigarette smoke could cause aging-like damage. Human lung fibroblasts exposed to cigarette smoke extract exhibited an aging-associated secretory phenotype, releasing a range of inflammatory cytokines and chemokines, thus fostering a proinflammatory milieu and encouraging immune cell recruitment [41]. In human bronchial epithelial cells exposed to cigarette smoke extract, there was an accumulation of mitochondrial fragments, reduced autophagy, heightened reactive oxygen species (ROS) generation, and cellular aging [53]. Smoke-exposed mice also exhibited similar patterns of impaired autophagy and cellular aging [54]. These findings clearly indicated that starting to smoke at an earlier age could lead to a larger pool of individuals being prone to ARDS. Additionally, our research revealed that cessation of smoking could not markedly lower this risk. This underlined the importance of focusing on early smoking prevention and cessation strategies to decrease ARDS risk, particularly in younger populations.

Measuring cigarette smoke exposure in epidemiological studies is challenging. Cohort studies evaluating the quantitative link between smoking and ARDS often measured tobacco metabolite levels (cotinine, NNAL) to identify exposed individuals [9, 15, 17, 18, 36, 55]. Previous studies indicated that in certain patient groups, such as those with blunt trauma injuries and nonlung sepsis, higher levels of tobacco product metabolites were identified as independent risk factors for ARDS [17, 18]. However, our meta-analysis indicated that the current thresholds for tobacco metabolite detection did not markedly elevate ARDS risk in identified smokers. In contrast, a noticeable increase in ARDS risk was observed in populations identified through inquiries about smoking history. These findings suggested that the existing thresholds for tobacco metabolite screening might be too low, including a large group with minimal exposure to cigarette smoke, which were not substantially affected. Therefore, revising these thresholds could lead to more accurate identification of high-risk ARDS patients in clinical practice.

The studies enrolled in our research did not provide data on e-cigarette vaping. Although e-cigarettes were often perceived as less harmful alternatives to traditional cigarettes, recent studies indicated that they could cause pulmonary inflammation, injury, and other pathological changes associated with ARDS [56, 57]. Similar to the traditional cigarettes, e-cigarette vaping could provoke inflammatory responses and oxidative stress, activating pulmonary macrophages and epithelial cells to release proinflammatory cytokines, leading to damage in alveolar epithelial and endothelial cells [58, 59]. Additionally, e-cigarette vaping might increase the risk of ARDS through unique mechanisms, such as toxic metabolites from flavorings and harmful compounds produced at high temperatures, causing cytotoxic responses and alveolar death [57]. Consequently, e-cigarette vaping might elevate the risk of ARDS. Future clinical research should consider e-cigarette vaping among patients to clarify its relationship with the incidence of ARDS.

This study had several limitations. Notably, our results exhibited high heterogeneity. We did extensive subgroup and sensitivity analyses to explain this issue. The results showed that clinical risk factors, assessment methods of smoking status, and ARDS diagnostic criteria were not the primary contributors to the observed heterogeneity. The inherent heterogeneity might be attributed to the characteristics of the observational studies, which often contained uncontrolled confounding factors. Regrettably, available information was too limited for further exploration. Future large-scale prospective studies were recommended to validate these findings. Additionally, the possibility of publication bias was considered, yet the statistical analysis suggested it did not affect the stability of our results. Moreover, two case–control studies linking smoking with a higher risk of transfusion-related lung injury were not included in our meta-analysis due to incomplete data [60, 61]. Finally, given the limited number of studies, the results should be interpreted with caution. Updated meta-analyses incorporating future research findings were recommended to provide a more comprehensive understanding of the topic.

## Conclusion

In conclusion, this meta-analysis contributed further evidence that smoking increases the risk of ARDS.

### Supplementary Information

**Additional file 1: Tab. S1.** PRISMA Checklist. **Tab. S2.** Search strategy. **Tab. S3.** The sensitivity analysis, comparing clinical risk factors for ARDS. **Fig. S1**. Subgroup analysis, comparing clinical risk factors, **Fig. S2**. The funnel plot and results of the trim and fill analysis. **Fig. S3**. The sensitivity analysis. **Fig. S4**. Subgroup analysis, comparing ARDS diagnostic criteria. **Fig. S5**. Subgroup analysis, comparing smoking status assessment methods.

## Data Availability

All the data associated with this manuscript were included in the main text and supplementary materials.

## Abbreviations

ARDS: Acute respiratory distress syndrome
ALI: Acute lung injury
NNAL: 4-(Methylnitrosamino)-1-(3-pyridyl)-1-butanol
CI: Confidence interval
MD: Mean difference
ISS: Injury severity score
OR: Odds ratio
AECC: American-European Consensus Conference definition
ICD-10: The International Classification of Disease-10
APACHE II: Acute physiology and chronic health evaluation II
NF-κB: Nuclear factor kappa-light-chain-enhancer of activated B cells
ROS: Reactive oxygen species

## References

[1] Bos LDJ, Ware LB (2022). Acute respiratory distress syndrome: causes, pathophysiology, and phenotypes. Lancet.

[2] Bellani G, Laffey JG, Pham T, Fan E, Brochard L, Esteban A, Gattinoni L, van Haren F, Larsson A, McAuley DF (2016). Epidemiology, patterns of care, and mortality for patients with acute respiratory distress syndrome in intensive care units in 50 countries. JAMA.

[3] Meyer NJ, Gattinoni L, Calfee CS (2021). Acute respiratory distress syndrome. Lancet.

[4] Sheu CC, Gong MN, Zhai R, Chen F, Bajwa EK, Clardy PF, Gallagher DC, Thompson BT, Christiani DC (2010). Clinical characteristics and outcomes of sepsis-related versus non-sepsis-related ARDS. Chest.

[5] Reilly JP, Christie JD (2015). Primed for injury: cigarette smokers and acute respiratory distress syndrome. Crit Care Med.

[6] Christenson JT, Aeberhard JM, Badel P, Pepcak F, Maurice J, Simonet F, Velebit V, Schmuziger M (1996). Adult respiratory distress syndrome after cardiac surgery. Cardiovasc Surg.

[7] Moazed F, Calfee CS (2014). Environmental risk factors for acute respiratory distress syndrome. Clin Chest Med.

[8] Iribarren C, Jacobs DR, Sidney S, Gross MD, Eisner MD (2000). Cigarette smoking, alcohol consumption, and risk of ARDS: a 15-year cohort study in a managed care setting. Chest.

[9] Calfee CS, Matthay MA, Eisner MD, Benowitz N, Call M, Pittet J-F, Cohen MJ (2011). Active and passive cigarette smoking and acute lung injury after severe blunt trauma. Am J Respir Crit Care Med.

[10] Gajic O, Dabbagh O, Park PK, Adesanya A, Chang SY, Hou P, Anderson H, Hoth JJ, Mikkelsen ME, Gentile NT (2011). Early identification of patients at risk of acute lung injury: evaluation of lung injury prediction score in a multicenter cohort study. Am J Respir Crit Care Med.

[11] Goniewicz ML, Eisner MD, Lazcano-Ponce E, Zielinska-Danch W, Koszowski B, Sobczak A, Havel C, Jacob P, Benowitz NL (2011). Comparison of urine cotinine and the tobacco-specific nitrosamine metabolite 4-(methylnitrosamino)-1-(3-pyridyl)-1-butanol (NNAL) and their ratio to discriminate active from passive smoking. Nicotine Tob Res.

[12] Gongola A, Bradshaw JC, Jin J, Jensen HK, Bhavaraju A, Margolick J, Sexton KW, Robertson R, Kalkwarf KJ (2021). Retrospective study on rib fractures: smoking and alcohol matter for mortality and complications. Trauma Surg Acute Care Open.

[13] Dai B, Kang J (2010). Wang Z-f, Kong D-l, Tan W, Zhao H-w: [Risk factors of novel severe influenza A(H1N1) with concurrent adult respiratory distress syndrome]. Zhonghua Yi Xue Za Zhi.

[14] Aakre BM, Efem RI, Wilson GA, Kor DJ, Eisenach JH (2014). Postoperative acute respiratory distress syndrome in patients with previous exposure to bleomycin. Mayo Clin Proc.

[15] Calfee CS, Matthay MA, Kangelaris KN, Siew ED, Janz DR, Bernard GR, May AK, Jacob P, Havel C, Benowitz NL (2015). Cigarette smoke exposure and the acute respiratory distress syndrome. Crit Care Med.

[16] Rachmawati E, Listiowati E, Kurniawan DW, Suraya I, Ahsan A, Nurmansyah MI (2021). Significance of chronic diseases and smoking behavior in the development of acute respiratory distress syndrome among hospitalized COVID-19 patients in Indonesia. Asia Pac J Public Health.

[17] Moazed F, Hendrickson C, Conroy A, Kornblith LZ, Benowitz NL, Delucchi K, Cohen MJ, Calfee CS (2020). Cigarette smoking and ARDS after blunt trauma: the influence of changing smoking patterns and resuscitation practices. Chest.

[18] Moazed F, Hendrickson C, Jauregui A, Gotts J, Conroy A, Delucchi K, Zhuo H, Arambulo M, Vessel K, Ke S (2022). Cigarette smoke exposure and acute respiratory distress syndrome in sepsis: epidemiology, clinical features, and biologic markers. Am J Respir Crit Care Med.

[19] Lei H, Minghao W, Xiaonan Y, Ping X, Ziqi L, Qing X (2013). Acute lung injury in patients with severe acute pancreatitis. Turk J Gastroenterol.

[20] Iriyama H, Abe T, Kushimoto S, Fujishima S, Ogura H, Shiraishi A, Saitoh D, Mayumi T, Naito T, Komori A (2020). Risk modifiers of acute respiratory distress syndrome in patients with non-pulmonary sepsis: a retrospective analysis of the FORECAST study. J Intensive Care.

[21] Elmer J, Hou P, Wilcox SR, Chang Y, Schreiber H, Okechukwu I, Pontes-Neto O, Bajwa E, Hess DR, Avery L (2013). Acute respiratory distress syndrome after spontaneous intracerebral hemorrhage. Crit Care Med.

[22] Ying J, Zhou D, Gu T, Huang J (2019). Endocan, a risk factor for developing acute respiratory distress syndrome among severe pneumonia patients. Can Respir J.

[23] Afshar M, Netzer G, Mosier MJ, Cooper RS, Adams W, Burnham EL, Kovacs EJ, Durazo-Arvizu R, Kliethermes S (2017). The contributing risk of tobacco use for ARDS development in burn-injured adults with inhalation injury. Respir Care.

[24] Lassen MCH, Skaarup KG, Sengeløv M, Iversen K, Ulrik CS, Jensen JUS, Biering-Sørensen T (2021). Alcohol consumption and the risk of acute respiratory distress syndrome in COVID-19. Ann Am Thorac Soc.

[25] Wacharasint P, Fuengfoo P, Rangsin R, Morakul S, Chittawattanarat K, Chaiwat O (2016). Hazards and intensive care unit economic burden of cigarette smoking on critically Ill surgical patients: analysis of the THAI-SICU study. J Med Assoc Thai.

[26] Balfanz P, Hartmann B, Muller-Wieland D, Kleines M, Hackl D, Kossack N, Kersten A, Cornelissen C, Muller T, Daher A (2021). Early risk markers for severe clinical course and fatal outcome in German patients with COVID-19. PLoS ONE.

[27] Howells PA, Aldridge KA, Parekh D, Park D, Tucker O, Dancer RCA, Gao F, Perkins GD, Thickett DR (2017). ARDS following oesophagectomy: a comparison of two trials. BMJ Open Respir Res.

[28] Sang S, Sun R, Coquet J, Carmichael H, Seto T, Hernandez-Boussard T (2021). Learning from past respiratory infections to predict COVID-19 outcomes: retrospective study. J Med Internet Res.

[29] Li S, Zhao D, Cui J, Wang L, Ma X, Li Y (2020). Prevalence, potential risk factors and mortality rates of acute respiratory distress syndrome in Chinese patients with sepsis. J Int Med Res.

[30] Ferro TN, Goslar PW, Romanovsky AA, Petersen SR (2010). Smoking in trauma patients: the effects on the incidence of sepsis, respiratory failure, organ failure, and mortality. J Trauma.

[31] Chen W, Liu L, Yang J, Wang Y (2020). MicroRNA-146b correlates with decreased acute respiratory distress syndrome risk, reduced disease severity, and lower 28-day mortality in sepsis patients. J Clin Lab Anal.

[32] Huang X, Zhao M (2019). High expression of long non-coding RNA MALAT1 correlates with raised acute respiratory distress syndrome risk, disease severity, and increased mortality in sepstic patients. Int J Clin Exp Pathol.

[33] Matusov Y, Li J, Resuello D, Mathers H, Fried JC (2020). Use of pressure-regulated volume control in the first 48 hours of hospitalization of mechanically ventilated patients with sepsis or septic shock, with or without ARDS. J Intensive Care Soc.

[34] Wang Y, Fu X, Yu B, Ai F (2019). Long non-coding RNA THRIL predicts increased acute respiratory distress syndrome risk and positively correlates with disease severity, inflammation, and mortality in sepsis patients. J Clin Lab Anal.

[35] Shi Y, Wang L, Yu S, Ma X, Li X (2022). Risk factors for acute respiratory distress syndrome in sepsis patients: a retrospective study from a tertiary hospital in China. BMC Pulm Med.

[36] Panzer AR, Lynch SV, Langelier C, Christie JD, McCauley K, Nelson M, Cheung CK, Benowitz NL, Cohen MJ, Calfee CS (2018). Lung microbiota is related to smoking status and to development of acute respiratory distress syndrome in critically Ill trauma patients. Am J Respir Crit Care Med..

[37] Xu L, Li X, Wang H, Xie F, Liu H, Xie J (2019). Cigarette smoke triggers inflammation mediated by autophagy in BEAS-2B cells. Ecotoxicol Environ Saf.

[38] Lugg ST, Scott A, Parekh D, Naidu B, Thickett DR (2022). Cigarette smoke exposure and alveolar macrophages: mechanisms for lung disease. Thorax.

[39] Marwick JA, Kirkham P, Gilmour PS, Donaldson K, MacNee W, Rahman I (2002). Cigarette smoke-induced oxidative stress and TGF-beta1 increase p21waf1/cip1 expression in alveolar epithelial cells. Ann N Y Acad Sci.

[40] Zhang Y, Huang W, Zheng Z, Wang W, Yuan Y, Hong Q, Lin J, Li X, Meng Y (2021). Cigarette smoke-inactivated SIRT1 promotes autophagy-dependent senescence of alveolar epithelial type 2 cells to induce pulmonary fibrosis. Free Radical Biol Med.

[41] Li L, Yang DC, Chen CH (2021). Metabolic reprogramming: a driver of cigarette smoke-induced inflammatory lung diseases. Free Radic Biol Med.

[42] Sopori M (2002). Effects of cigarette smoke on the immune system. Nat Rev Immunol.

[43] Liang GB, He ZH (2019). Animal models of emphysema. Chin Med J (Engl).

[44] Mutepe ND, Cockeran R, Steel HC, Theron AJ, Mitchell TJ, Feldman C, Anderson R (2013). Effects of cigarette smoke condensate on pneumococcal biofilm formation and pneumolysin. Eur Respir J.

[45] Münzel T, Hahad O, Kuntic M, Keaney JF, Deanfield JE, Daiber A (2020). Effects of tobacco cigarettes, e-cigarettes, and waterpipe smoking on endothelial function and clinical outcomes. Eur Heart J.

[46] Shin I-S, Shin N-R, Park J-W, Jeon C-M, Hong J-M, Kwon O-K, Kim J-S, Lee I-C, Kim J-C, Oh S-R (2015). Melatonin attenuates neutrophil inflammation and mucus secretion in cigarette smoke-induced chronic obstructive pulmonary diseases via the suppression of Erk-Sp1 signaling. J Pineal Res.

[47] Kaminski TW, Brzoska T, Li X, Vats R, Katoch O, Dubey RK, Bagale K, Watkins SC, McVerry BJ, Pradhan-Sundd T (2024). Lung microvascular occlusion by platelet-rich neutrophil-platelet aggregates promotes cigarette smoke-induced severe flu. JCI Insight.

[48] Rocco PRM, Pelosi P (2008). Pulmonary and extrapulmonary acute respiratory distress syndrome: Myth or reality?. Curr Opin Crit Care.

[49] Hsieh SJ, Zhuo H, Benowitz NL, Thompson BT, Liu KD, Matthay MA, Calfee CS (2014). Prevalence and impact of active and passive cigarette smoking in acute respiratory distress syndrome. Crit Care Med.

[50] Hinojosa E, Boyd AR, Orihuela CJ (2009). Age-associated inflammation and toll-like receptor dysfunction prime the lungs for pneumococcal pneumonia. J Infect Dis.

[51] Sapey E, Greenwood H, Walton G, Mann E, Love A, Aaronson N, Insall RH, Stockley RA, Lord JM (2014). Phosphoinositide 3-kinase inhibition restores neutrophil accuracy in the elderly: toward targeted treatments for immunosenescence. Blood.

[52] Schouten LRA, Schultz MJ, van Kaam AH, Juffermans NP, Bos AP, Wösten-van Asperen RM (2015). Association between maturation and aging and pulmonary responses in animal models of lung injury: a systematic review. Anesthesiology.

[53] Ito S, Araya J, Kurita Y, Kobayashi K, Takasaka N, Yoshida M, Hara H, Minagawa S, Wakui H, Fujii S (2015). PARK2-mediated mitophagy is involved in regulation of HBEC senescence in COPD pathogenesis. Autophagy.

[54] Wang Y, Liu J, Zhou J-S, Huang H-Q, Li Z-Y, Xu X-C, Lai T-W, Hu Y, Zhou H-B, Chen H-P (2018). MTOR suppresses cigarette smoke-induced epithelial cell death and airway inflammation in chronic obstructive pulmonary disease. J Immunol.

[55] Moazed F, Hendrickson C, Nelson M, Conroy A, Cohen MJ, Calfee CS (2018). Platelet aggregation after blunt trauma is associated with the acute respiratory distress syndrome and altered by cigarette smoke exposure. J Trauma Acute Care Surg.

[56] Park JA, Crotty Alexander LE, Christiani DC (2022). Vaping and lung inflammation and injury. Annu Rev Physiol.

[57] Marrocco A, Singh D, Christiani DC, Demokritou P (2022). E-cigarette vaping associated acute lung injury (EVALI): state of science and future research needs. Crit Rev Toxicol.

[58] Glynos C, Bibli S-I, Katsaounou P, Pavlidou A, Magkou C, Karavana V, Topouzis S, Kalomenidis I, Zakynthinos S, Papapetropoulos A (2018). Comparison of the effects of e-cigarette vapor with cigarette smoke on lung function and inflammation in mice. Am J Physiol Lung Cell Mol Physiol.

[59] Serpa GL, Renton ND, Lee N, Crane MJ, Jamieson AM (2020). Electronic nicotine delivery system aerosol-induced cell death and dysfunction in macrophages and lung epithelial cells. Am J Respir Cell Mol Biol.

[60] Toy P, Bacchetti P, Grimes B, Gajic O, Murphy EL, Winters JL, Gropper MA, Hubmayr RD, Matthay MA, Wilson G (2015). Recipient clinical risk factors predominate in possible transfusion-related acute lung injury. Transfusion.

[61] Sun L, Liu Y (2022). Clinical factors of blood transfusion-related acute lung injury and changes in levels of treg-related cytokines. Emerg Med Int.

